# The Myth of Normal Reading

**DOI:** 10.1177/17456916221127226

**Published:** 2022-11-10

**Authors:** Falk Huettig, Fernanda Ferreira

**Affiliations:** 1 Psychology of Language Department, Max Planck Institute for Psycholinguistics, Nijmegen, the Netherlands; 2 Department of Language and Communication, Radboud University Nijmegen; 3 Department of Psychology, University of California, Davis

**Keywords:** dyslexia, literacy, reading, reading disorders

## Abstract

We argue that the educational and psychological sciences must embrace the diversity of reading rather than chase the phantom of normal reading behavior. We critically discuss the research practice of asking participants in experiments to read “normally.” We then draw attention to the large cross-cultural and linguistic diversity around the world and consider the enormous diversity of reading situations and goals. Finally, we observe that people bring a huge diversity of brains and experiences to the reading task. This leads to four implications: First, there are important lessons for how to conduct psycholinguistic experiments; second, we need to move beyond Anglocentric reading research and produce models of reading that reflect the large cross-cultural diversity of languages and types of writing systems; third, we must acknowledge that there are multiple ways of reading and reasons for reading, and none of them is normal or better or a “gold standard”; and fourth, we must stop stigmatizing individuals who read differently and for different reasons, and there should be increased focus on teaching the ability to extract information relevant to the person’s goals. What is important is not how well people decode written language and how fast people read but what people comprehend given their own stated goals.

The way society determines what is “normal” and “abnormal” and the implications of that distinction have received increased critical attention in recent years. The *APA Dictionary of Psychology* (American Psychological Association, n.d.) defines normal as “relating to what is considered standard, average, typical, or healthy.” Here we deconstruct the myth of normal reading. In doing so we argue that there is no such thing as standard, average, typical, or healthy reading and that the concept of normal reading is best confined to the dustbin of history in the educational and psychological sciences. Finally, we discuss the implications of this view and provide recommendations for reading researchers and practitioners.

## Psycholinguistic Studies of Normal Reading

First, we consider the issue that psycholinguistic studies typically aim to investigate normal reading and that participants are often even instructed to read normally in experiments. We argue that this makes little sense given the diversity of the backgrounds of participants and the fact that outside the Anglo-Saxon world multilingualism is the norm rather than the exception (Romaine, 2017). Either implicitly or explicitly, the fields of psycholinguistics and language development have generally assumed that there is something called “normal reading” (as evident from the titles of many journal articles; e.g., Aghababian & Nazir, 2000; Horwitz et al., 1998; Kennedy & Pynte, 2005; Staub et al., 2009) and that it is largely the topic we wish to study.

When it comes to the materials participants in psycholinguistic experiments are asked to process, it is difficult to imagine that those readers are deeply engaged in trying to grasp the nuances of meaning or attempting to relate the contents to what they already know in any elaborative way. A sentence such as “The evidence examined by the lawyer turned out to be unreliable” (typical for psycholinguistic experiments; e.g., Ferreira & Clifton, 1986) probably means almost nothing to the participant in the absence of information about what the evidence is, who is being tried, what the court case is, and so on. Moreover, Christianson et al. (2022) made a convincing case that many participants in our experiments are not motivated to read such sentences in a “typical manner.” At the same time, readers who are participants in experiments probably know they cannot get away with simply skimming the words or sentences because sometimes (not often enough) they will be asked some sort of “comprehension question” to determine whether they have achieved some typically not well-defined level of comprehension.

Rather than being able to assume the participant is engaged in normal reading, we should acknowledge that participants are reading in a very special and atypical mode: They are trying to get enough details and gist to answer simple comprehension tests about items they care little about. This reading mode might be appropriate and sufficient for answering many important psycholinguistic questions, and therefore we do not recommend abandoning its use (indeed, we rely on it in our own work). What we do believe is that the field should cease calling this normal reading, because even if we knew what normal reading was, what is taking place in our experiments is almost certainly not it.

When it comes to the participants, the adult college undergraduate is a stand-in for this normal reader, who is treated as coming into the lab to perform in an experiment with essentially the same lexicon, grammar, and reading skills as the experimenter and the scientists who devised the experiment—the typical hearer/speaker. This “normal subject” is asked to read what is usually a series of unrelated words or sentences, one after another, and to proceed through this list in a way that seems intuitively appropriate—that is, they are asked to “read normally,” a concept that is left up to the participant to unpack and implement. A quick survey of articles published in preferred psycholinguistic outlets shows that a large number of them specifically invoke the concept of normal reading in their descriptions of instructions to participants. The instruction to read normally is presumably meant to encourage participants to ignore the artificial reading materials and the rather strange lab situation and read as if they were in a less artificial reading situation. The reading mode itself, however, is not specified in the participant instructions and will almost inevitably be interpreted differently by different participants. Moreover, merely instructing participants to read normally does not make the reading situation any less artificial.

Some may consider this to be an innocent set of practices because they are predicated on the ideas that there is a normal language user, that the college undergraduate represents that reader well, and that there is a default mode for processing language that can be thought of as normal as opposed to involving some special strategy. Careful scrutiny of each idea, however, calls the entire approach into question. We challenge each one in turn.

## Diversity of Brains and Experiences

The idea of a normal language user raises several critical questions. One fundamental question on which there is little consensus is whether the normal reader should be monolingual to avoid potential contaminating effects from an uncontrolled set of other languages, or whether it is normal to know and use more than one language. Some experimental standards regarding what types of participants should serve as controls in reading research, for example, are problematic. These standards could be argued to be the legacy of an often unquestioned history of examining reading in American and British monolingual communities in the latter part of the 20th century. It is noteworthy that almost every article on second-language acquisition includes a group of monolingual subjects to serve as controls, but almost no research focusing on monolingual reading includes bilingual or multilingual control groups. Some studies appeal to the notion of a native speaker instead of requiring monolingualism, but that status is typically self-defined, and many studies now question its scientific basis and utility (Doerr, 2009; Hackert, 2012; Phillipson, 2016), particularly because we know that what someone considers their native language versus their dominant language often diverges. Another critical question is the age of the participant. Standard practice in adult psycholinguistic studies is to exclude people who are under the age of 18, but it is unclear whether this legal definition captures the range of abilities that one sees in adolescents, and the lack of any official upper bound on age ignores a large body of research showing significant differences in language-processing strategies between young adults and those over the age of 60, and sometimes even younger (Miller & Miller Stine-Morrow, 1998; Miller et al., 2004; Payne et al., 2014; Stine-Morrow et al., 2000).

The notion that college undergraduates are good stand-ins for this questionable idea of the normal reader is also problematic (Ashby et al., 2005). Contrary to our stereotype of college students, undergraduates come from a wide range of backgrounds and have had varied linguistic experiences (Matsuda, 2006). They do not all speak the same dialect of the language under study, and they therefore may have different vocabularies and grammatical systems. Some of them will have been diagnosed with a language-related condition at some point prior to arriving at college, and it is not known how those conditions might still affect adult language processing, including ideas or strategies they may have learned from learning or speech therapists. Many of them enroll in college classes with currently active, diagnosed conditions that affect their cognitive and language skills, including dyslexia, autism, and attention-deficit/hyperactivity disorder. Some researchers might question whether someone who fits the diagnostic criteria of dyslexia can represent the so-called normal reader, but most labs lack procedures for identifying such individuals and excluding them from studies (Lopes et al., 2020). More importantly, if our research is to truly reflect the diversity of human minds and linguistic experience, then it could be argued that it is inappropriate to exclude this segment of the population from our experiments. Unfortunately, at this stage we cannot settle this question; our point, however, is to note the importance of the issue and the lack of any serious discussion about it.

In short, it is apparent that people bring a huge diversity of brains and experiences to the reading task. We stress that neurodivergence and individual differences are all part of a continuum that make up successful reading behaviors (Andrews, 2015). Readers, ranging from low literates to highly proficient and almost speed readers, use a spectrum of reading styles and strategies, and it makes little sense to label any of them abnormal. A case in hand is that most people who have reading disorders such as dyslexia read and succeed in extracting useful information.

## Diversity of Reading Situations and Goal

There is enormous diversity of reading situations and goals. People read for many purposes. They read for pleasure, skim for information, read physics textbooks, bring different amounts of knowledge to bear on a topic, and so on. People do not read the same way across all of these different situations and with the diverse goals they have in mind (Radach et al., 2008).

If we consider psycholinguistic experiments, and if the issues discussed above were unproblematic (i.e., even if we could assume we know what a normal reader is and we agreed that we have good reason to believe college undergraduates fall into that category), the idea concerning what takes place during experiments remains, and it is in some ways even more questionable. The fact that even in published psycholinguistic studies people show very different reading rates suggests that participants read quite differently (even when taking the different reading materials into account). Brysbaert (2019) reported that average word reading rates in the Nelson-Denny Reading Test (Brown et al., 1993; Nelson & Denny, 1929), in which participants were asked to read at their “normal” rate, were around 250 words per minute (wpm); in the Chapman-Cook Speed of Reading Test, average word reading rates were around 300 wpm; in the Michigan Speed of Reading Test, average word reading rates were around 212 wpm; and in the Minnesota Speed of Reading Test, average word reading rates were 154 wpm when tested in 1928 and 130 wpm when tested in 1978 (Eurich & Kraetsch, 1982).

Skilled readers such as the people reading this piece read in a variety of different ways depending on their goals, and none of those ways seems inherently abnormal. We read for entertainment and to extract complex information. We sometimes skim a piece of writing because we are looking for a specific fact or we have read something similar before. We might read a love letter or an email expressing displeasure with something we said or did with a great deal of focus and attention, making every effort to relate the words in the missive to relevant experiences and knowledge.

Reading is affected by the properties of the written media. We read differently depending on whether the setting is a formal or informal social one and whether the medium is print or online (Foasberg, 2014). Even when reading online we may read somewhat differently on a desktop computer than a smartphone. Many people do not have access to a desktop computer for online reading, and so texts and resources have to be read on very small smartphone screens. Reading is affected by properties of the writing genres. We read differently depending on whether we read romance or comedy, crime and mystery, or speculative fiction such as fantasy, science fiction, and horror. We may read action and historical fiction differently from nonfiction such as biography and autobiography or self-help and popular science. Again, none of these ways of reading seems abnormal; what matters is whether the approach makes sense given the nature of the text and the goals of the reader.

## Cross-Cultural and Linguistic Diversity

There are many different types of writing systems in use across the world, and there is a huge diversity in the properties they exhibit (for discussion, see Daniels, 2021). Alphabets (e.g., English writing) use characters for most of the individual segments, including vocalic and consonantal phonemes. Syllabaries (e.g., Vai in Liberia) use characters for each syllable. Logosyllabaries (e.g., Chinese) use logograms for their syllabic and semantic values. Augmented alphabets (e.g., Coptic) use characters for individual segments as well as some syllables. Abjads (e.g., Arabic, Hebrew) use characters for consonants only (vowels have to be inferred by the reader). Abugidas (e.g., Devanagari used to write Hindi) use characters to encode a consonant with an inherent vowel and diacritics modifying the vowel. Abugidas therefore encode syllabic and subsyllabic information simultaneously. It is important to realize that even within these main script categories there is huge diversity. Alphabetic writing systems, for instance, can be orthographically transparent (e.g., Italian) or orthographically opaque (e.g., English). Characters in abugidas can transcend syllabic boundaries. For the Hindi word *namaste* there is a character denoting the sequence “-ste,” but the syllables are na-mas-te. Furthermore, reading the same language in a different writing system has very different demands on the reader. Reading Mandarin Chinese in Hanyu Pinyin places very different demands on the reader than reading simplified or traditional Chinese characters. Reading Urdu in Persian script is different from reading Hindi in Devanagari (although both are more or less the same language). Persian typically does not encode vowels but Devanagari does, and so on. Reading a language that makes use of one type of writing system (e.g., English) is very different from reading a language uses several intermingled types of writing systems. Reading Japanese typically involves four intermixed writing systems: syllabic Hiragana and Katakana, logographic Kanji, and the occasional alphabetic Romaji. Even reading a language that makes use of one type of writing system such as Portuguese or French with lots of diacritics is somewhat different from reading in a language such as English that tends not to use them.

The diversity of writing systems makes it apparent that English is an outlier in terms of both linguistic properties and orthography. This state of affairs is augmented by the fact that spoken languages also differ in many different ways and that these differences ultimately affect how languages are read independently of the type writing system they are written in. This has the consequence that findings from the reading of English do not necessarily generalize and are not necessarily relevant across the diversity of writing systems found in the world. This may sound like an obvious or trivial point, yet the Anglocentrism of a lot of reading research remains a significant problem (Share, 2008).

## Conclusion

We conclude that the educational and psychological sciences must embrace the diversity of reading rather than chase the phantom of normal reading behavior and fight reading wars about the better method of reading instruction (Pearson, 2004). This leads to certain implications.

### Implication 1: conducting ecologically valid psycholinguistic experiments of reading

The first implication is that psycholinguists should reconsider the widespread practice of asking participants to read normally in experiments meant to investigate either psycholinguistic questions (e.g., syntactic processing) or basic processes in reading (e.g., text characteristics that trigger regressive eye movements). As we have argued, given the diversity of potential experimental participants as well as the diversity of the goals and assumptions they bring to the laboratory, it is probably unwise to assume they understand what we believe normal reading to be and that they know how to implement that standard. A better practice would be to eschew the concept of normality entirely and instead state specifically how the researchers would like the participants to approach the text, with several examples. Depending on the goals of the study, participants might be asked to read each sentence in such a way that they could paraphrase its meaning immediately, or after two to three other sentences had been read. A different instruction might be to warn the participants that immediately after some of the sentences they will be asked a true/false question that can be easily answered correctly as long as each word of the sentence is read. Yet another instruction might be to read so that a challenging question that requires drawing some type of pragmatic inference about the sentence can be answered correctly. What is critical is to (a) avoid the use of the term “normal” for the reasons we have described here; (b) inform the participants of the type of question or probe that they will be given, and how frequently; (c) specify whether the question or probe will follow immediately or after some specified number of intervening items; and (d) include several examples so that the participant knows exactly what level of comprehension is desired.

One major benefit of adopting this set of practices is that doing so would improve the reproducibility of psycholinguistic research. A problematic side effect of relying on intuitive notions of normal reading and of not providing participants with instructions as detailed as what we have suggested here is that each lab will instruct their participants somewhat differently and in ways that have not typically been well documented. A lab at a large public university in the American Midwest at which researchers ask their undergraduate participants to “read normally” will likely elicit a different standard of reading behavior than will a lab located at an elite university in a bilingual European country or an underresourced lab in an Asian country. These differences may be at least in part responsible for some of the discrepancies in results that have been observed in the reading and psycholinguistics literature and over which there is often intense theoretical disagreement.

Of course, our argument is not that the artificiality of many reading experiments implies that the results from such studies are uninformative; some of the artificiality is, after all, a consequence of the need to control for potentially confounding variables. What needs to be established, however, is whether the findings from laboratory studies that invoke this idea of normal reading generalize to more naturalistic reading situations and task settings. It is high time that reading research starts to systematically explore the effects of different reading situations, styles, materials, and task goals. It may well turn out that some aspects of the reading process are quite stable, whereas others, we suspect, are more malleable.

### Implication 2: moving beyond Anglocentric reading research

The second implication is that we need to move truly beyond Anglocentric reading research and produce theoretical and computational models of reading that reflect the large cross-cultural diversity of languages and types of writing systems. Progress in the science of reading will be substantially hindered if it continues to be the case that the vast majority of studies are carried out in English. The controversy about access to morphological information during parafoveal processing in reading is a case in point. Morphological information refers to knowledge of the internal structure of a word, including its root and any affixes. According to the original E-Z reader model, perhaps the most influential model of eye movements in reading and developed originally mostly on data from English, morphological and semantic information is not processed parafoveally (Rayner et al., 2003; Schotter et al., 2012). However, studies of reading in other writing systems, such as the one used for Hebrew (Deutsch et al., 2005), Russian (Stoops & Christianson, 2017), and Chinese (Yen et al., 2008), strongly suggest that morphological information can be accessed from parafoveal preview. If the data that motivate theories are derived primarily from a handful of languages from the same linguistic family, it is impossible to know whether the conclusions apply generally or only to those specific cases.

Share (2021b) has recently evaluated the response of the field to his 2008 *Psychological Bulletin* (Share, 2008) request that research must reflect better the reality of global diversity of languages and writing systems. His assessment of the current state of affairs makes for depressing reading. Acknowledging limited progress he points out that reading research remains very much entrenched in Anglocentrism and that the dominant theories of cross-script diversity, orthographic depth, and psycholinguistic grain-size theory largely ignore non-European alphabets or nonalphabetic scripts. Indeed, it is very much apparent from the published literature that most academic reading research appears not to deem it necessary to specify the language under investigation in the title of empirical studies of reading. In our experience as journal editors it is not unusual for reviewers to question the need for reading research in other languages or writing systems on the grounds that “it has already been shown in English.” We embrace the conclusion of Share (2021a) that “if the science of reading is to contribute meaningfully to assessment, diagnosis, instruction, and intervention for all readers around the world, then we must extricate our field from entrenched ethnocentrism and embrace global diversity.”

### Implication 3: acknowledging and supporting the diversity of ways of reading

The third implication is to acknowledge that there are multiple ways of reading and reasons for reading, none of which are normal or better or a “gold standard” for how people should read. As far as reading is concerned, it is not true that one size fits all.

People’s level of reading experience varies a lot, and those factors will influence how we approach reading and what an episode of reading is for. We must take to heart that we read for different purposes: novels versus journal articles versus menus versus Twitter feeds versus directions for taking medicine. What is normal depends on the situation and the task at hand (Lim & Christianson, 2015). People read very differently from when they read the same article the very first time than when they read it a second time. Reading for pleasure is also diverse; some people like spoilers, whereas others do not. When reading someone who writes engaging stories but also writes beautifully, readers sometimes appear to force themselves to slow down to take in the quality of the prose. Reading for pleasure, coupled with deep engagement, is thus likely to lead to pretty careful decoding—that is, deeper than the satisfactory processing that takes place in other situations. In many other cases, it may be different; some form of “good enough word decoding” (cf. Ferreira & Patson, 2007) may be sufficient and quite normal; for example, because of the inconsistency and irregularity of English orthography, L2 readers and people with reading disorders may ignore some of the “spelling” as long as meaning is retrieved. And that is just as well.

There are many reading modes, all of which have their advantages and disadvantages with regard to specific reading goals and the intended level of coherence. Readers are often able to efficiently execute different reading modes that are “optimal” in a specific reading situation. The construct of normal reading, however, implies more than this: It implies that there is a normative standard that applies to all reading situations. Although it might not be true that the construct of normal reading means one way is optimal or better, it certainly does imply a standard against which others are judged and must be described as not normal. We agree that there are many different reading modes that have advantages and disadvantages with respect to the reader’s goals, and we agree that readers adopt the mode that suits their task. However, we also believe these assumptions can be made without invoking the problematic standard of normal reading.

### Implication 4a: stopping stigmatizing individuals who read differently

The fourth implication is twofold and concerns how we consider individual differences. For one we need to stop stigmatizing individuals who read for different reasons and who read differently.

More than 30 different types of developmental dyslexia, each resulting from a breakdown at a different stage of the reading process, have been reported (Friedmann & Coltheart, 2016). Even if we acknowledge that a phonological deficit is a valid proximal explanation of dyslexia (Share, 2021a), the underlying distal causes of reading disorders are still largely unidentified. The implications of this, which have not fully reached all clinical and educational practitioners and even researchers, is that there is no single cause of dyslexia and no single cure. People with dyslexia improve with extensive training mostly because they learn to develop certain strategies to minimize the impact of their reading disorder. People with reading disorders often work harder and get additional training; read more slowly and allow themselves more time for a given text; use line trackers or color overlays; read aloud; keep logs or diaries of essential words; reread words, sentences, or whole passages; write in different colors; and make extensive use of computer software to check their spelling. As a strategy, forms of prediction might help to overcome decoding difficulties. We should stop stigmatizing individuals (Gibbs & Elliott, 2020) who rely on guessing to get around word-decoding difficulties for example. All of these strategies help people with dyslexia to function in society, and some people get so sophisticated in these procedures that outsiders may hardly notice any signs of reading difficulty.

We must accommodate people’s different cognitive reading machinery. We must stop labeling children dyslexic in 1st or 2nd grade. Such labels stick, affect children’s confidence, and are counterproductive and misplaced. Another controversial practice that must be confined to the dustbin of history is to identify reading difficulties on the basis of a discrepancy between reading and cognitive abilities (as in the definition of the International Dyslexia Association [IDA], “often unexpected in relation to other cognitive abilities,” IDA, n.d.). This is a particularly worrisome practice because it tends to discriminate against readers from lower socioeconomic backgrounds. Classification criteria that are often interpreted as relating to intelligence defined by a score on an IQ test do not even provide important information about the nature of the reading difficulty (Siegel & Hurford, 2019). Definitions of dyslexia also tend to discriminate against children who are reading poorly for other reasons or who are learning to read in their nonnative language. As a consequence, these children often do not get the educational support they require.

We emphasize that in some situations it is useful to make some normative statements about reading: That is, if the goal of reading is X but the reader in question does not read in a way that will allow X to be achieved, that is indeed problematic. Again, however, our point is not that there are no benchmarks in different situations but that scientists and educators should avoid the use of terms such as “normal” and the baggage they carry. People with dyslexia might indeed wish to learn to read in a way that allows them to read many texts the way their peers do, and helping them to achieve that goal is of course an important endeavor. But this goal likely can be achieved without appealing to the concept of normality.

### Implication 4b: radically increased focus on reading comprehension when teaching reading

The final point concerns teaching methods of how reading is taught and the infamous “reading wars” (Castles et al., 2018; Pearson, 2004). It is a matter of fact that around the world reading is taught in different ways; some countries focus more on phonics than others, even among alphabetic languages. We certainly do not question, for example, the usefulness of teaching phonics to beginning readers of alphabetic writing systems. Decoding abilities necessarily predict some comprehension abilities in beginning readers of alphabetic scripts, so teaching decoding makes obvious sense. We strongly believe, however, that there should be increased focus on the teaching of *comprehension* of written materials and critical literacy (Morais, 2018; Morais & Kolinsky, 2021; White & Cooper, 2015) and the ability to extract information that is relevant to the person’s goals, not as measured against some abstract benchmarks of reading performance.

Reading-comprehension abilities appear to be in decline across the globe (Organisation for Economic Co-operation and Development, 2019). In Belgium, for example, a country with “traditionally” relatively high literacy levels, less than 10% of 15-year-old students achieve a PISA reading proficiency level 5 or above (which is equivalent to high reading comprehension). These are quite dramatic and worrying numbers. Declining literacy is likely to have substantial negative socioeconomic impact. To respond to these trends appropriately it is pertinent to devise effective and efficient measures to improve literacy. In important ways, educational policies and the science of reading arguably have gotten it all wrong: The focus should not be so much on how well people decode written language and how fast people read but instead on what people comprehend given their own stated goals (cf. Graesser et al., 1994; Kaakinen et al., 2002; Snow, 2002).

## Transparency

*Action Editor:* Timothy J. Pleskac

*Editor:* Klaus Fiedler
